# Unlocking the Rich Potential of a Soft Gel-Cream Enriched with Royal Jelly for Topical Use

**DOI:** 10.3390/gels11040294

**Published:** 2025-04-16

**Authors:** Monica-Elisabeta Maxim, Raluca-Marieta Toma, Ludmila Aricov, Anca-Ruxandra Leonties, Aurica Precupas, Rodica Tatia, Elena Iulia Oprita

**Affiliations:** 1 Romanian Academy, Ilie Murgulescu—Institute of Physical Chemistry, 202 Splaiul Independentei, 060021 Bucharest, Romania; mmaxim@icf.ro (M.-E.M.); laricov@icf.ro (L.A.); aleonties@icf.ro (A.-R.L.); aprecupas@icf.ro (A.P.); 2 National Institute of Research and Development for Biological Sciences, 296 Splaiul Independentei, 060031 Bucharest, Romania; rodica.tatia@incdsb.ro (R.T.); iulia.oprita@incdsb.ro (E.I.O.)

**Keywords:** royal jelly, sodium hyaluronate, soft gel-cream, characterization, cytotoxicity

## Abstract

For decades, royal jelly achieved notoriety and became an ultra-rich ingredient with numerous pharmacological properties especially for its use in production of topical ointments and creams. A novel formulation enriched with 2% royal jelly has been developed and characterized. Rheological results highlight a gel-like behavior of the product in the packaging, as it does not flow from the costumer’s hand after application and behaves like a liquid, spreading evenly onto clean skin. A clear comparison in size distribution of pure and cream samples was noticed by dynamic light scattering analysis and completed further by Fourier transform infrared spectroscopy (FTIR-ATR) which showed off shift changes in the gel sample as compared to pure compounds. MTT assays were conducted in quintuplicate on murine fibroblasts cell line (NCTC L-929) for testing the biocompatibility of the product in the range of 50–1000 μg/mL over 24, 48 and 72 h. The designed formulation is typically intended to deliver active compounds to the skin surface and potentially into deeper layers. A molecular docking study was performed for binding mode prediction of P-gp protein residues with two ligands, quercetin and myricetin, in order to investigate their role in the internal modulation of drug transport across cell membranes within the skin.

## 1. Introduction

Many bee-derived products such as honey, royal jelly and propolis have been widely used in health and cosmeceutical industries since decades [1,2]. Royal jelly (RJ) represents a unique honey bee compound, high in nutrients with complex intrinsic attributes for the organism [3]. Besides the rich content of crude proteins, sugars and vitamins, RJ contains small amounts of flavanols and flavanones such as apigenin, quercetin or naringenin previous reported for pronounced ability to scavenge free radicals [4,5]. RJ is well-known for its anti-inflammatory properties [6] and for enhancing collagen production [7]. Bioactive molecules are already known for their therapeutic effects in many conditions [8]. It is not new to the academic community that natural products efficiently modulating the activity of P-gp (P-Glycoprotein) [9], a transmembrane protein for optimizing drug delivery and cell targeting [10]. Particularly, these compounds could be represented by quercetin and myricetin, two plant-derived flavonoids also found in bee products [11] and recognized for their nutraceutical properties and numerous health benefits [12,13]. Topical formulations are directly related to the properties of the carrier using specific transport vehicles [14]. The permeability of the significant molecule through the various layers of the skin is modulated by the constituents of the carrier (e.g., therapeutic drugs) [15]. Molecular docking studies of protein–ligand complexes are essential for optimization, development and topical delivery of various small active molecules, providing information on binding affinities at target sites for future understanding, designing and developing of pharmaceutics [16].

Sodium hyaluronate (NaHA) represents the sodium salt of hyaluronic acid, a natural polymer used frequently in skin formulations on the strength of water molecules binding capacity [17]. Research has demonstrated that NaHA’s biological activity in hydrating the outermost epidermal layer by drawing moisture from the dermis is molecular weight-dependent [18,19,20]. Therefore, high molecular weight NaHA manifests anti-inflammatory properties and its rheological behavior determine its usage for topical preparations [21,22]. Emollient agents create protective films on the skin surfaces [23]. Shea butter and almond oil represent few examples of skin emollients used in cosmetology due to their contribution to the skin-barrier potential because of the high essential fatty acids content [24], which enhances the permeability processes [25] and being integral components of the cellular mediated transport [26].

However, little has been reported in the literature regarding the in vitro evaluation of the potential proliferative effect of a gel-cream enriched with RJ. Therefore, we selected for the presented study, RJ, as a key component in the development of an own soft-gel formulation for topical use. Rheology provided valuable insights into the viscoelastic behavior of the gel-cream sample correlated to its spreading mode on the skin. Fourier transform infrared spectroscopy (FTIR-ATR) showed structural features and characteristic absorption bands in the cream sample and pure components. Dynamic light scattering underlined comparative analysis of intensity distributions of cream and neat samples. MTT assays were conducted in quintuplicate on NCTC L-929 murine fibroblast cell line and showed proliferative effect of the tested cream concentrations in the first 24 h at 250 and 500 μg/mL. Biological tests were further succeeded by insights into molecular docking of the complexes made by amino acids residues of P-gp transmembrane protein and two small ligands, quercetin and myricetin, in order to explore the binding affinities and bond patterns for future understanding of skin delivery processes.

## 2. Results and Discussion

### 2.1. Synthesis and Evaluation of RJ-Based Formulation

**Figure 1:**
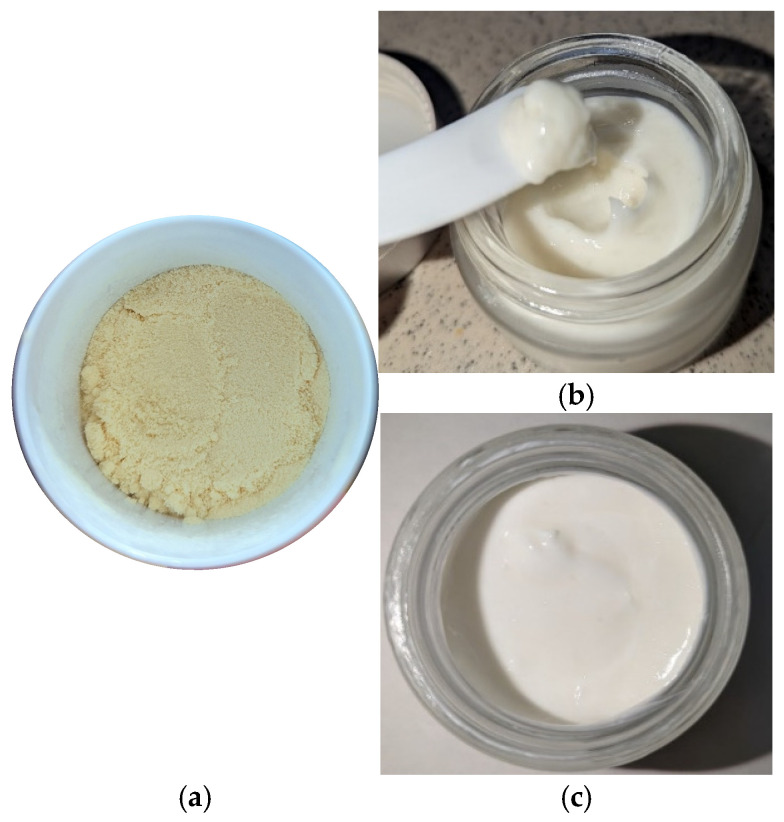
(**a**) RJ organic powder; (**b**,**c**) RJ-based soft gel-cream (50 mg of product).

A promising topical product with nourishing properties based on natural ingredients was synthetized and characterized (Figure 1a–c). The main ingredients and dosages (%) used in preparation of the gel-cream are shown in Table 1 and Table 2 below. The formulation showed good consistency and a fine homogeneous texture without any sediments and phase separations in time. Moreover, it was distinguished by a discreet floral scent and a milky white color. The product was neither greasy nor sticky after water removal with an emollient effect, optimal for topical use [27]. The emulsifying agent (Olliva) contains cetearyl and sorbitan olivate, which effectively promotes the delivery of water-soluble actives at the level of the epidermis [28]. RJ (CAS 8031–67-2, https://ec.europa.eu/growth/tools-databases/cosing/details/79870, accessed on 13 April 2025) organic powder purchased from Elemental SRL Company is certified as an organic farming product according to European Pharmacopoeia, EU regulation 2023/2652 and COSMOS standard (https://www.cosmos-standard.org/en/databases/, accessed on 13 April 2025) used in topical formulations in the range 1–4%. Recent studies showed that the incorporation of 1% RJ had a high influence on the hydration level of the outermost layer of the epidermis [29]. Topical products containing RJ should have an optimal pH in the range of 4 to 6 [30]. The pH of fresh RJ usually ranges between 3.6 and 4.2 [31]. The pH value of the gel-cream is situated in the range between 5 and 6, according to other similar topical formulations [30].

**Figure 2:**
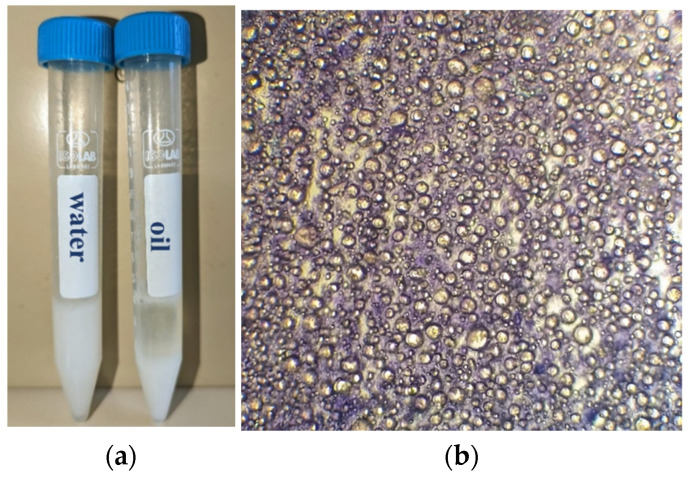
(**a**) Dilution test with 3 mL of water and oil, respectively. (**b**) Dye test under microscope at 10× magnification with Methylene Blue 0.1%.

The product was kept in a glass container and storage at room temperature for further experimental investigations. The saponification (SV) and acid values (AV) were determined in triplicate according to an Asian protocol for oil analysis [32,33] adapted to the current research. The obtained value for SV was 212.265 mg KOH/g sample and 6.75 mg KOH/g sample for the AV, as stated by other papers with similarities regarding the obtained values of raw materials [34,35,36]. A dilution test with both water and oil is represented in the Figure 2a. In the first case, no phase is changed when water is added to the sample, but secondly a phase break occurs when oil is added. The dilution test was succeeded by a dye test. A small amount of gel-cream sample was deposited on a microscopic slide and examined with a 10× magnification in order to identify its pattern. As is observed in Figure 2b, lipid globules appear colorless under a dark violet background, which highlights the formation of a water continuous phase with oil droplets.

### 2.2. Scattering Measurements

**Figure 3:**
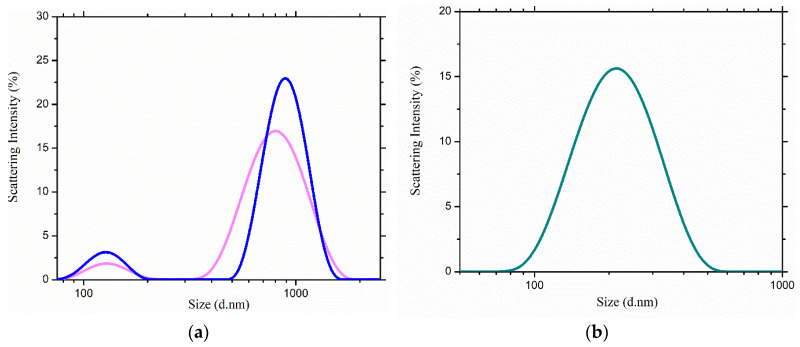
(**a**) Size distribution by intensity of (**a**) RJ (light magenta) and RJ-NaHA (blue) systems; (**b**) gel-cream sample (turquoise).

DLS experiments were conducted to explore the size distributions of neat and soft gel-cream samples in aqueous medium. The concentrations of the stock solutions were related to the dosages (%) exposed in Table 2, reported to a final volume of 5 mL for each sample, as follows: 2% RJ (*w/v*), 2% RJ (*w/v*) with 0.4% NaHA (*w/v*) and 1% emulsion gel (*w/v*). From that, 10 μL of each stock solution was added to 2990 μL water, homogenized with continuous magnetic stirring and further analyzed via scattering measurements. A recent study showed a peak shift induction in size distributions, when human norovirus particles were incubated and treated with a natural extract based on RJ, indicating particle aggregation [37]. Similarities were observed in the case of RJ-NaHA distribution presented below. Figure 3a shows a slower diffusion of the particles thence wide populations were obtained with an average of 792.42 nm in the case of free RJ and 888.18 nm for the RJ-NaHA sample with the additional presence of two small peaks with close values at 127.5 and 130 nm, respectively.

RJ represents a sophisticated chemical with a high content of vitamins, proteins, (un)saturated linear fatty acids and, last but not least, polyphenols [38]. As a consequence of its complexity, the size distributions of neat samples are related to possible hydrogen bonding interactions. Due to the simultaneous presence of hydrophilic portions in the polymeric chain and oleic or stearic hydrophobic portions of lipid tails, hyaluronan molecules showed a tendency to create nanoaggregates in aqueous solution [39], explained by a slight increase in the intensity in the case of RJ-NaHA sample. The dispersed phase contains oil droplets whose sizes are critically important. The distribution of the gel-cream sample is presented in the Figure 3b. The scattering profile is corelated to a monomodal distribution of small particles in suspension, lower in intensity compared to neat samples. The distribution of the formulation is characterized by a measured size of 210.82 nm. Each experiment was performed three times and representative measurements are presented above.

### 2.3. FTIR-ATR

The FTIR-ATR method was used to characterize the gel-cream and its active components as follows: NaHA, which provides a good hydration of the skin and RJ essential for restoration of the epidermis and fading of wrinkles. NaHA spectrum shows a broad band at 3277.28 cm^−1^ corresponding to O-H bond and the peak at 2889.06 cm^−1^ is related to C-H stretching vibration. In accordance to Gili et al. and Pan et al., the bands at 1605.41 cm^−1^ and 1404.57 cm^−1^ are assigned to asymmetric (C=O) and symmetric (C-O) stretching vibration of the planar carboxyl deprotonated groups characteristic of NaHA [40,41,42]. The peak at 1319.45 cm^−1^ is assigned to NH group (amide III). A small peak at 1147.56 cm^−1^ confirms the presence of C-O-C group (O-bridge) corresponding to polysaccharide structure and a strong peak at 1030.55 cm^−1^ confirms the presence of C-OH groups [43]. Two small peaks at 946.54 cm^−1^ and at 894.87 cm^−1^ might indicate the asymmetric out-of-phase ring vibration and δ (C1-H) vibrational mode specific to β-hexopyranoses [41], respectively. In the RJ spectrum, a broad band centered at 3276.43 cm^−1^ is associated with the stretching vibration of O-H from water and N-H stretching vibration of primary and secondary amines [44,45]. Two peaks at 2928.49 cm^−1^ and 2857.16 cm^−1^ correspond to the C-H stretching vibration in the methylene groups. The absorption bands in the region of 1700–1500 cm^−1^ are related to the protein molecular structure. Thus, the strong absorption band at 1646.33 cm^−1^ can be attributed to the C=O stretching vibration in amide I groups (α-helix structure) and the absorption band at 1539.82 cm^−1^ corresponds to the coupled C-N stretching vibration and N-H bending vibrations of peptide bonds (amide II groups) [45,46]. The region between 1400 and 1200 cm^−1^ is assigned to amide III groups. The strong appearance at 1023.53 cm^−1^ is related to C-O bond from associated hydroxyl or phenol groups and correlate to the presence of 10-hydroxy-2-decenoic acid (10-H_2_DA), gluconic acid and polyphenols [47].

**Figure 4:**
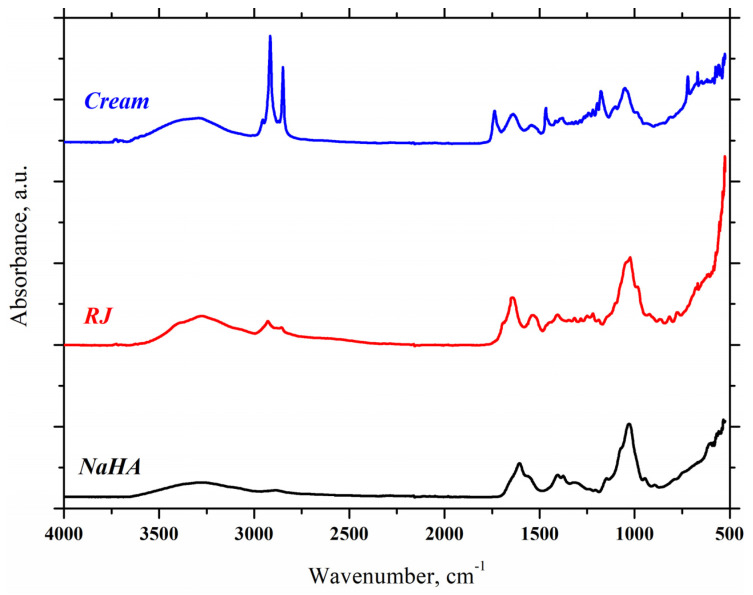
FTIR-ATR absorbance spectra of gel-cream sample and pure compounds.

The FTIR-ATR spectrum of moisturizing formulation reveals a broad band resulted from O-H and N-H overlapping of stretching vibration centered at 3292.08 cm^−1^ (Figure 4). The peaks in the region between 2950 and 2849 cm^−1^ are assigned to both symmetric and asymmetric stretching vibration of C-H bond in methylene and methyl groups. A sharp peak at 1736.50 cm^−1^ can be attributed to C=O stretching vibration in carbonyl functional group of esters and confirm the presence of almond oil, olive oil and shea butter with saturated fatty acids content. The band at 1637.22 cm^−1^ is due to C=O stretching vibration in amide I group (from RJ proteins) [44], and also may be related to isolated water from emulsion gel. At the same time the band at 1541.31 cm^−1^ is attributed to C-N stretching vibration and N-H bending vibrations of amide II groups from RJ components. Two absorption bands at 1466.71cm^−1^ and at 1378.92 cm^−1^ are assigned to C-H deformation vibration and bending vibrations of the methyl groups, respectively [48,49]. A well-defined peak at 1177.57 cm^−1^ is assigned to C-O stretching vibrations of ester bond and a strong peak at 1050.71 cm^−1^ is assigned to the C-O bond from associated hydroxyls or phenol groups in hyaluronic acid, RJ components and rose water.

### 2.4. Rheological Properties

**Figure 5:**
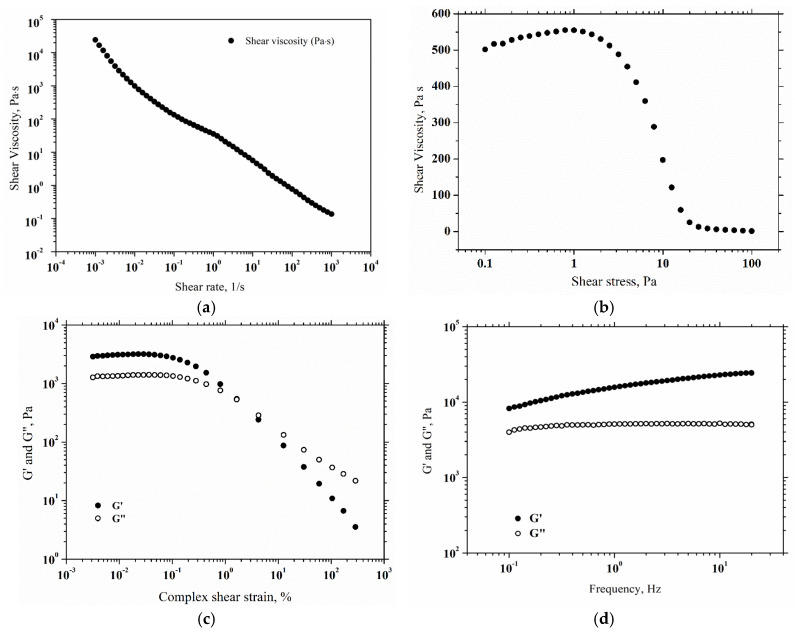
(**a**) Viscosity variation with shear rate; (**b**) viscosity variation with shear stress; (**c**) elastic and viscous moduli (G’ and G”) variation with shear strain; (**d**) elastic and viscous moduli (G’ and G”) variation with frequency (amount of product needed to perform rheological tests was 30 mg).

Rheology represents a very important part of the next steps in handling and using topical creams, such as applying, mixing, storing and pumping [50]. The relationship between viscosity and applied shear rates in the range 10^−^^3^–10^3^ s^−^^1^ at 25 °C is presented in Figure 5a. The viscosity curve of the product showed a shear thinning behavior, as the viscosity decreases with shear rate across the whole examined range. When the applied shear rate is low, the soft gel is very close to being at rest, and its behavior is reflected in higher viscosity. This result guarantees that the gel will not flow from the packaging without external forces and, once applied to the skin, it will adhere to the skin without running off [51]. When the cream is subjected to higher shear rates, a reduction in the product’s viscosity is observed, thinning the product. The behavior of the cream during its spread on the skin is also mirrored by the higher shear rates [52]. Also, the low viscosity facilitates this process, resulting in a feeling of smoothness on the epidermis. Moreover, the yield stress or the minimum stress required to cause a material to flow, is an important parameter [53]. As a result, the yield stress measurement on the prepared cream is presented in Figure 5b. The sample structure stretching before yielding causes an apparent increase in viscosity in the lower shear stress range. Following the viscosity peak, the product begins to flow. At a shear stress of 1.007 Pa, the cream starts to flow, marking the yielding point. Furthermore, the amplitude shear strain results at a frequency of 1 Hz and a temperature of 25 °C are presented in Figure 5c. Elastic modulus (G’) and viscous modulus (G”) showed linear viscoelastic behavior up to 0.1% strain, followed by decreasing pattern of G’ and G”. The limit of the linear viscoelastic region is marked by the decrease in rheological moduli with shear strain, indicating irreversible deformation of the hydrogel-cream. Figure 5d presents the viscoelastic properties of the cream. The shear strain of 0.05% was applied based on the prior amplitude sweep tests, so that the shear strain is in the linear viscoelastic region. Thus, G’ and G” were measured in the frequency range of 0.1–20 Hz. Over all frequency ranges, the elastic modulus was higher than the viscous modulus. This viscoelastic response suggests a gel-like behavior of the cream. In conclusion, the prepared cream behaves like a gel when it is in the packaging and does not flow from the consumer’s hand after being distributed on the skin, then behaves like a liquid during spreading on the skin.

### 2.5. Molecular Docking Study

Various therapeutic agents or active compounds delivered across the skin are known to be substrates of P-Glycoprotein (P-gp) [54]. These include immunosuppressive drugs, antibiotics, antiviral drugs and corticosteroids such as tacrolimus, erythromycin, acyclovir, dexamethasone, prednisolone and betamethasone [55,56,57,58] for topical application, and opioid analgesics, psychotropic drug, beta-blockers and estrogens such as fentanyl, buprenorphine, methylphenidate, bisoprolol and estradiol [59,60,61,62] for transdermal delivery. P-gp mediates active secretion of its substrate drugs in liver and kidney [63,64] and is responsible for active efflux of its substrate drugs across brain endothelial cells and small intestinal epithelial cells [65,66]. P-glycoprotein (P-gp), a plasma membrane protein that confers multidrug resistance on cells due to its ability to exclude cytotoxic drugs, has been identified in epidermal keratinocytes of both human and mouse [67,68,69]. The endogenous and xenobiotic compounds are transported across membranes against the concentration gradient. The P-gp structure includes three drug-binding sites within the transmembrane cavity: the H site for Hoechst 33342 binding, the R site for rhodamine 123 binding [70], and a modulator-binding site (M site) [71]. Also, ATP-binding and subsequent hydrolysis sites present on both the NBD domains have been reported and assigned in the P-gp structure. It has been investigated the potential binding interactions between P-gp with quercetin, the main polyphenol in RJ, and myricetin, a molecule with similar structure and an additional hydroxyl group grafted on ring B, respectively. We analyzed and parametrized both molecules for a complete overview of a topical administration of a soft gel-cream with 2% RJ. Few studies highlighted that quercetin and myricetin showed inhibitory effects on P-gp activity, mostly localized at the substrate-binding site [72].

**Figure 6:**
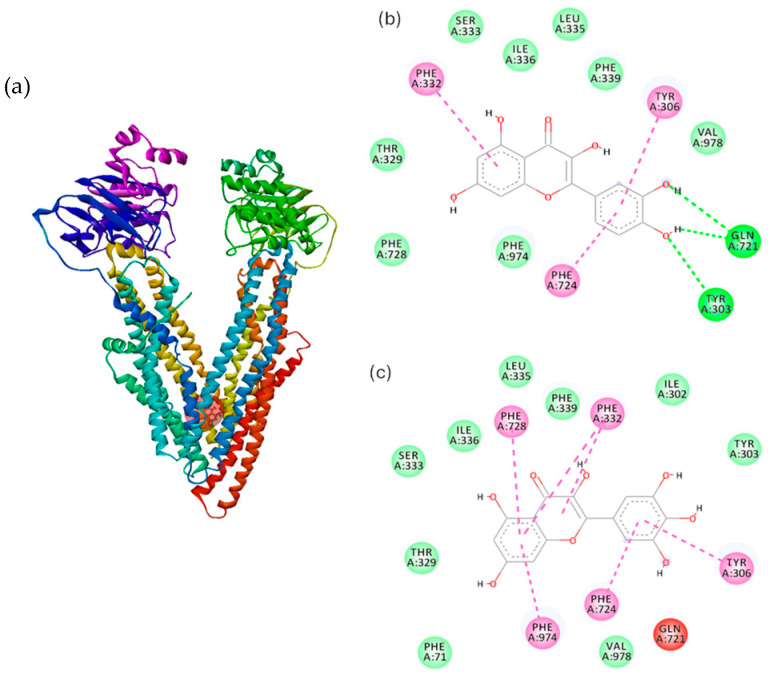
Molecular docking of quercetin and myricetin with P-glycoprotein. (**a**) Quercetin is presented in pink ball and stick model, myricetin in green ball and stick model, and the protein as solid ribbon; (**b**) 2D representation of quercetin–P-gp complex, (**c**) 2D representation of myricetin–P-gp complex; close amino acid residues are presented in green, dashed lines represent intermolecular interactions of different origin (hydrogen bonds—green lines, hydrophobic—magenta lines).

Hence, molecular docking of quercetin and myricetin with P-gp was performed in order to identify the binding mode and the interacting forces for the ligand-protein complex. As shown in Figure 6a, the binding sites for quercetin and myricetin with P-gp are similar, in the hydrophobic transmembrane domain (TMD). The amino acids involved in the interaction are presented in the 2D plots in Figure 6b–c and the nature of interacting forces is presented in Table 3, where are highlighted the major interactions between ligands and the protein residues as follows: hydrogen bonds, van der Waals and hydrophobic interactions. The value of quercetin binding affinity is similar to that obtained for quercetin binding to drug-binding site of human P-gp (−7.269 kcal/mol) as reported previously [72].

Docking analysis revealed hydrogen and hydrophobic interactions of quercetin and myricetin with the amino acid residues established in the binding pocket of P-gp; the hydrogen bonds of quercetin were found to be 2.83 Å for the residue TYR303 (OH…O), and 3.09 Å for GL721 (OE1…H). Moreover, π-π stacked interaction is located between π-orbitals of PHE724 and π-orbitals of quercetin, while π-π T-shaped interactions occur between π-orbitals of TYR 306, PHE 332 and π-orbitals of quercetin, respectively. Hydrophobic π-π stacked interactions were identified between π-orbitals of PHE724, PHE974 and π-orbitals of myricetin, respectively. π-π T-shaped interactions occur between π-orbitals of TYR 306, PHE 332, PHE728 and π-orbitals of myricetin, respectively. Bai et al. suggested that the inhibitory effect of flavonoids might be related to π interactions [73]. Quercetin performs as a P-gp modulator via impeding signal transduction from nucleotide-binding domain to transmembrane domain (TMD) [72,74]. The designed soft gel-cream formulation is typically intended to deliver active compounds to the skin surface and potentially into deeper layers. RJ’s lipid-rich content can enhance the skin penetration of hydrophobic molecules, improving their solubility, absorption, and bioavailability. Understanding the P-gp inhibitory potential of these flavonoids is important for targeting deeper skin layers: even though P-gp is not directly involved in skin penetration, quercetin and myricetin’s P-gp inhibition could play a huge role in the internal modulation of drug transport across cell membranes within the skin. This might ensure that more of the active ingredients reach their target tissues. P-gp inhibition could potentially increase the local concentration of other compounds used in conjunction with RJ, making them more effective at lower doses. Quercetin was reported to be the most effective P-gp inhibitor among the other flavonoids, while myricetin presents moderate inhibitory activity [75]. Furthermore, while P-gp inhibition is more relevant in oral formulations, the potential to enhance the effectiveness of topical treatments via enhanced absorption makes this formulation promising for skin care applications.

Hence, docking results suggest that quercetin and myricetin can bind to hydrophobic regions of P-gp, and in the context of topical formulations, this may help in overcoming any efflux transport mechanisms in the skin cells, leading to more efficient drug delivery to targeted skin layers (e.g., dermis or epidermis).

### 2.6. Cytotoxicity Assay

**Figure 7:**
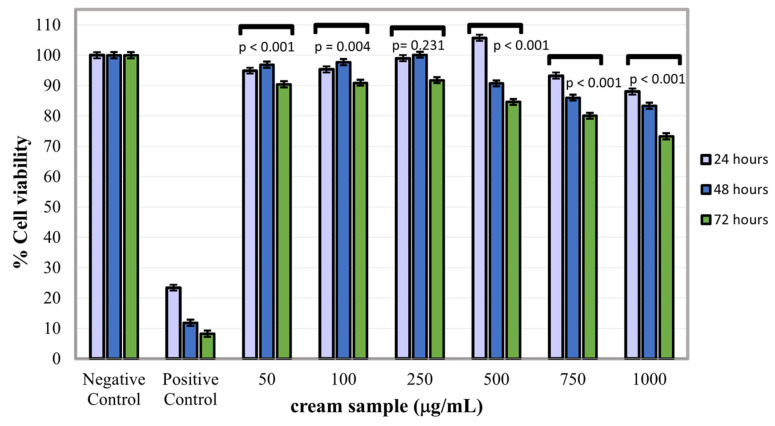
MTT assay after 24, 48 and 72 h of treatment. Error bars indicate standard deviations. One-way analyses of variance (ANOVA) was performed and *p*-values are related to specific groups.

The MTT assay of the gel-cream sample at all the three-time intervals, in the concentration range of 50–1000 µg/mL, indicated that the sample extract manifested biocompatibility with NCTC fibroblasts, recording cell viability values of 80–105.70%, with the exception at the maximum concentration of 1000 µg/mL at 72 h of treatment, when the formulation extract induced a slightly cytotoxic effect (73.29% cell viability) on fibroblast cells (Figure 7). The experimental results indicated that the highest fibroblast proliferation rate (105.70%) was induced by the gel-cream extract at a concentration of 500 µg/mL at 24 h of treatment, and also at 250 µg/mL at 48 h of treatment, with 100.14% viability. Other studies showed remarkable abilities of RJ powder such as inducing collagen synthesis and cell proliferation observed both on normal human skin fibroblasts and ventricular cells from neonatal rats [76,77,78]. Also, a correlation was observed between the testing concentration of the sample and the rate of induced cell proliferation. Data obtained from the in vitro cytotoxicity evaluation of the colloidal gel extract at concentrations range of 50–1000 µg/mL using the MTT spectrophotometric method of quantifying the proportion of live cells in the NCTC culture la 24, 48 and 72 h of treatment, respectively, have indicated good biocompatibility of the sample with NCTC normal fibroblasts. Hence, cell proliferation values of NCTC fibroblasts after the three treatment time intervals was dependent on sample concentration which showed a very good biocompatibility.

## 3. Conclusions

A topical RJ-based soft gel-cream has been formulated, characterized and biological tested. The product was non-greasy and safe in respect to skin irritation protocol. The acid and saponification values were related to a high content of fatty acids found in almond oil, shea butter and emulsifier compositions. Rheology showed an excellent viscoelastic behavior of the soft formulation in the applied friction forces. The Fourier transform infrared spectroscopy (FTIR-ATR) investigated the vibrational states and spectral shifts occurred in pure and product samples. Moreover, scattering measurements gave us a comparison of the intensity distributions in neat and gel samples. MTT assays showed very good biocompatible results for the tested product concentrations. At the concentration of 500 µg/mL at 24 h of treatment, a high proliferation rate of 105.70% was observed. Moreover, at 250 µg/mL at 48 h of treatment, the proliferation rate had a value of 100.14%. Furthermore, cytotoxicity assays were preceded by additional molecular docking investigation of P-glycoprotein binding with two similar polyphenols, quercetin and myricetin, through major hydrophobic interactions for additionally molecular mechanisms of action. The approached methods conferred a complete overview for a topical soft gel-based on RJ.

## 4. Materials and Methods

### 4.1. Materials

Royal jelly (RJ) organic powder, high molecular weight hyaluronic acid sodium salt (HMW-NaHA) (1000–1600 kDa), tocopherol (vitamin E), organic shea butter, almond oil, o/w emulsifying agent (based on cetearyl olivate and sorbitan olivate), rose hydrosol, sweet violet fragrance and preservative (cosgard) were purchased from Elemental SRL company (Oradea, Romania) specialized in the distribution sale of active and natural ingredients for cosmetic formulation use only. Origin of products can be found in Table 2, Section 2.2. All products were certified by Ecocert Greenlife according to COSMOS-standard regulations and were used without further purification.

### 4.2. Preparation and Evaluation of RJ-Soft Gel-Cream

The oil-soluble components were dissolved in the oil phase (Part A containing shea butter, almond oil and Olliva emulsifier) and gently heated up to 75 °C in the bain-marie on a plate with magnetic stirring at 200 rpm. The water components (ultrapure water and rose hydrosol) were dissolved in the aqueous phase (Part B) and heated also to 75 °C. After heating, the oil phase was added to the water phase and thereafter, the beaker was introduced in a cool water bowl and the phases were mixed using a T25 digital Ultra-Turrax Ika disperser (Germany) until the texture of the soft formulation was homogenous and uniform. Below 40 °C, other ingredients such as royal jelly, NaHA, tocopherol, fragrance and preservative (Part C) were added and thoroughly blended until smooth. Hence, the product was kept in a glass container and examined after 24 h. The dosages (%) for obtaining 50 mg of product are exposed in Table 2, Section 2.2. The gel-cream was further characterized and biological tested. The physical appearance of the colloidal system was observed by its color, smell and texture. The pH was determined at room temperature by taking and applying a tiny amount of sample on a pH-indicator strip. The product was tested further for the homogeneity by visual appearance and by touch. The ease of removal of the gel-cream applied was examined by washing the applied part with tap water. The sample was applied on dorsum of right hand and the application time was noted for any skin rashes.

### 4.3. Determination of Saponification and Acid Values

For saponification value (SV) determination, 0.30 mg of gel-cream was refluxed with 10 mL of 0.5 N alcoholic KOH for 30 min; to this, 2–3 drops of thymolphthalein was added and titrated immediately with a 0.5 N HCl solution until blue color disappeared. The saponification value (SV) was calculated according to the Equation (1):

SV = 56.11 × 0.5 × (Vs − Vb) × F/(sample weight)(1)

where Vs = titration volume of the sample (mL), Vb = titration volume of the blank (mL) and F = factor of 0.5 N HCl standard solution.

Similarly to the determinations above, acid value (AV) was obtained as follows: 0.3 mg of sample was dissolved in 10 mL of n-hexane and the flask was connected to reflux condenser and slowly heated until sample was dissolved completely; to this, 1–2 drops of thymolphthalein was added and titrated with 0.02 N alcoholic solution until a faintly blue color appeared after shaking for 30 s. The acid value (AV) was calculated in triplicate according to Equation (2):

AV = (56.11 × 0.02 × (Vs − Vb) × F)/(sample weight)(2)

where Vs = titration volume of sample (mL), Vb = titration volume of blank (mL) and F = factor of 0.02 N KOH solution. All the experiments were done in triplicate.

### 4.4. Identification Tests

During a dilution test, either oil or water is used to dilute the formed emulsion. The emulsion will remain stable if the water is the dispersion medium. 0.1 g of gel-cream sample was placed in two 15 mL plastic centrifuge tubes, in which 3 mL of oil or water were added and further examined. Moreover, Ceti Magnum-FL Trinocular Compound Microscope (Medline Scientific, Rotherham, UK) with LED Epi-Fluorescence Attachment was used for brightfield viewing of the sample. One drop of Methylene Blue 0.1% (*w*/*v*) was dusted on the surface of the tested sample placed on a microscopic slide, covered with a coverslip and examined it under microscope at 10× magnification. The images were captured by an attached Canon DSLR EOS 4000D (Canon, Tokyo, Japan) camera.

### 4.5. Dynamic Light Scattering (DLS)

The size distributions of the RJ and RJ-NaHA dispersed in neat water as well as for the gel-cream sample were performed with dynamic light scattering measurements conducted employing a Nano-ZS Sizer (Malvern Instruments Ltd., Malvern, UK) equipped with a HeNe red laser (λ = 633 nm) and operating at a scattering angle of 173 °C and at 25 °C.

### 4.6. Fourier Transform Infrared Spectroscopy (FTIR-ATR)

Fourier transform infrared spectroscopy (FTIR-ATR) spectra of the pure compounds (RJ, NaHA) and dried sample were recorded on a Nicolet i-S10 FTIR spectrometer (Thermo Scientific, Waltham, MA, USA) equipped with a diamond crystal and in the ATR mode in the 4000–600 cm^−1^ spectral region. The spectra were collected at 4 cm^−1^ resolution with 32 scans.

### 4.7. Rheology

A Kinexus Pro Rheometer (Malvern Panalytical, UK) was applied to evaluate the rheological behavior of the soft formulation. To maintain a temperature of 25 °C, a Julabo CF41 cryo-compact circulator was used. The rheometer was equipped with a geometry of 50 mm diameter and a gap of 0.6 mm. The shear rate, ranged from 10^−3^ to 10^3^ s^−1^, was used to measure the shear viscosity. Tests on amplitude sweep were performed between 0.1 and 1000% of shear strain at 1 Hz. The viscoelastic nature of the gel-cream was examined using a frequency sweep test (0.1 and 20 Hz) at a constant shear strain of 0.05%. All of the results were shown in logarithmic form.

### 4.8. Molecular Docking

The mechanism of drug modulation of P-gp has been extensively studied, both by experimental and computational methods. The flavonoid, quercetin, the most abundant polyphenol identified in the RJ composition, and myricetin, a compound with similar structure, were chosen for molecular docking with P-gp. The molecular docking studies of quercetin and myricetin with P-gp were performed by AutoDock Vina 1.1.2 software [79,80]. The mouse P-gp structure (PDB code: 4LSG, https://doi.org/10.2210/pdb4LSG/pdb, accessed on 13 April 2025) was obtained from the RCSB Protein Data Bank (available at http://www.rcsb.org; data was accessed on 3 April 2024). The geometry of the flavonoids was optimized without geometry constraints, using the functional B3LYP and the basis set 6–311 g(d,p), as previously described [81]. Density functional theory calculations were performed on the Gaussian 03 software [82]. The transmembrane domain of P-gp [83] was used to analyze the binding of the flavonoids, quercetin and myricetin, with P-gp. The non-polar hydrogen atoms were merged, and the polar hydrogen atoms were added using Autodock 4.2 Tools [84]. The protein structure was kept rigid during the docking, while the ligands were allowed to have rotatable bonds. The center of the grid box was set to x = 20, y = 52, z = −2 and the size was 40 × 38 × 36 in the x, y, z dimensions, with a grid point spacing of 1 Å, sufficiently large to include the H, M, and R binding sites. Molecular docking calculations were analyzed via Lamarckian Generic Algorithm [84]. The conformer with the lowest binding affinity was selected for analysis and the type of interaction was evaluated using the BIOVIA Discovery studio 2019 [85].

### 4.9. Cytotoxicity

The murine NCTC clone L-929 fibroblast cell line was purchased from ECACC (Sigma-Aldrich, Taufkirchen, Germany). The cytotoxicity of the sample was evaluated on a stabilized NCTC mouse fibroblast cell line at 24, 48 and 72 h of treatment using the cell viability MTT assay, carried out in accordance with the European standard SR EN ISO 10993–5:2009 (https://www.asro.ro/, accessed on 13 April 2025). In order to evaluate in vitro cytotoxicity of the gel- cream sample on NCTC fibroblasts, a stock solution was prepared by solubilization of the sample in a small amount of DMSO (dimethyl sulfoxide), followed by its dilution in culture medium until the concentration of 1000 µg/mL. For the experiment the cells were seeded in culture plates with 96 wells at a cell density of 4 × 104 cells/mL in MEM culture medium with 10% fetal bovine serum and 1% antibiotics, the plates being incubated at 37 °C in a humid atmosphere with 5% CO_2_. After 24 h of culture plates incubation, the culture medium was removed from the wells and over the cells was added stock solutions of the sample at concentrations of 50, 100, 250, 500, 750 and 1000 µg/mL, by dilution in culture medium. At 24, 48 and 72 h of treatment, the extracts in variable concentrations were replaced with a tetrazolium salt solution (5 mg/mL), and the experimental plate was incubated for 3 h under standard conditions, during which the specific reaction of the MTT assay generated formazan crystals in viable NCTC cells. After 3 h of incubation, the MTT solution was removed from the wells, the formazan crystals were solubilized by adding isopropanol, resulting in a blue-violet coloring of the solutions from the wells. The spectrophotometric measurement of the color intensity of the solutions was carried out using a Berthold Mithras UV-Vis LB940 spectrophotometer (Berthold Technologies, Bad Wildbad, Germany), at a wavelength of 570 nm. Untreated cells were considered as negative control of cell culture, while as positive control it was used a solution of 0.003% H_2_O_2_ in culture medium. The results were calculated as a percentage, based on untreated control cells, whose viability was considered 100%. Each of the experimental variants (concentrations or controls) was tested in five replicates.

## Figures and Tables

**Table 1:** Preparation of RJ-based soft gel-cream.

Phase A (Oil Phase > 70 °C)	Phase B (Water Phase > 70 °C)	Phase C (Humectants < 40 °C)
Shea butter	Ultrapure water	Royal jelly
Almond oil	Rose hydrosol	Sodium hyaluronate
Olliva emulsifier		Tocopherol
		*Viola Odorata* fragrance
		Preservative

*Note:* Active ingredients used in formulation.

**Table 2:** Key components, origins of ingredients and dosage (50 mg of product).

Components (INCI)	Country and Origin	Dosage (%)
Butyrospermum Parkii Butter	Burkina Faso, organic purified butter	8
*Prunus Amygdalus* Dulcis Oil	Germany, organic unrefined oil	12
Olliva Emulsifier (cetearyl olivate and sorbitan olivate)	Italy, natural origin	6
Water	Germany, Mili-Q water	50.5
Hydrolyzed *Rose Damascena* Flower extract	Bulgaria, organic farming	20
Royal jelly powder	France, organic farming	2
High molecular weight sodium hyaluronate	China, plant sources	0.4
Tocopherol, Helianthus Annuus seed oil	Belgium, natural origin,	0.5
*Viola Odorata* flower extract	France, natural fragrance oil	0.4
Cosgard (benzyl alcohol, salicylic acid, glycerin, sorbic acid, water)	USA, cosmetic preservative	0.6

*Note:* INCI (International Nomenclature of Cosmetic Ingredients).

**Table 3:** Molecular interactions between the two polyphenols and the amino acid residues in P-glycoprotein obtained by molecular docking.

Polyphenol	Amino Acid Residue	Distance, Å	Type of Interaction	Binding Affinity, kcal/mol
quercetin	TYR303	2.83	hydrogen bond	−7.6
	GLN721	3.09	hydrogen bond	
	PHE724	3.88	hydrophobic	
	TYR306	5.40	hydrophobic	
	PHE332	5.49	hydrophobic	
myricetin	PHE724	4.03	hydrophobic	−7.5
	PHE974	5.09	hydrophobic	
	TYR306	5.38	hydrophobic	
	PHE332	5.58	hydrophobic	
	PHE728	5.93	hydrophobic	

## Data Availability

The original contributions presented in this study are included in the article. Further inquiries can be directed to the corresponding author.
